# Sequence, Structure, and Binding Site Analysis of Kirkiin in Comparison with Ricin and Other Type 2 RIPs

**DOI:** 10.3390/toxins13120862

**Published:** 2021-12-03

**Authors:** Stefania Maiello, Rosario Iglesias, Letizia Polito, Lucía Citores, Massimo Bortolotti, José M. Ferreras, Andrea Bolognesi

**Affiliations:** 1 Department of Experimental, Diagnostic and Specialty Medicine—DIMES, Alma Mater Studiorum—University of Bologna, Via S. Giacomo 14, 40126 Bologna, Italy; stefania.maiello2@unibo.it (S.M.); massimo.bortolotti2@unibo.it (M.B.); andrea.bolognesi@unibo.it (A.B.); 2 Department of Biochemistry and Molecular Biology and Physiology, Faculty of Sciences, University of Valladolid, 47011 Valladolid, Spain; lucia.citores@uva.es (L.C.); josemiguel.ferreras@uva.es (J.M.F.)

**Keywords:** 3D structure, plant toxin, primary sequence, ribosome-inactivating protein, kirkiin, ricin, toxic lectin, sugar specificity, cancer therapy

## Abstract

Kirkiin is a new type 2 ribosome-inactivating protein (RIP) purified from the caudex of *Adenia kirkii* with a cytotoxicity compared to that of stenodactylin. The high toxicity of RIPs from *Adenia* genus plants makes them interesting tools for biotechnology and therapeutic applications, particularly in cancer therapy. The complete amino acid sequence and 3D structure prediction of kirkiin are here reported. Gene sequence analysis revealed that kirkiin is encoded by a 1572 bp open reading frame, corresponding to 524 amino acid residues, without introns. The amino acid sequence analysis showed a high degree of identity with other *Adenia* RIPs. The 3D structure of kirkiin preserves the overall folding of type 2 RIPs. The key amino acids of the active site, described for ricin and other RIPs, are also conserved in the kirkiin A chain. Sugar affinity studies and docking experiments revealed that both the 1α and 2γ sites of the kirkiin B chain exhibit binding activity toward lactose and D-galactose, being lower than ricin. The replacement of His246 in the kirkiin 2γ site instead of Tyr248 in ricin causes a different structure arrangement that could explain the lower sugar affinity of kirkiin with respect to ricin.

## 1. Introduction

Ribosome-inactivating proteins (RIPs) are plant toxic enzymes widely distributed in nature, and many RIP-containing plants have been used for centuries in traditional and folk medicine for the treatment of several pathologies [1,2]. Type 2 RIPs consist of two polypeptide chains, called A and B chain, which are linked together through a disulfide bridge. The A chain possesses rRNA N-glycosylase and polynucleotide:adenosine glycosidase activities that irreversibly damage rRNA and other polynucleotide substrates inside the cells, thus causing cell death [3]. The B chain has lectin properties, which allows type 2 RIPs to bind the galactoside residues on cell membrane, facilitating the entry into cells and resulting in high cytotoxicity.

Type 2 RIPs extracted from *Adenia* genus plants are the most potent plant toxins known to date, being able to irreversibly inhibit protein synthesis and to induce cell death at very low concentrations. In addition, *Adenia* RIPs proved very toxic for animals at small doses. This high toxicity is due to the peculiarity of *Adenia* toxins to be transported in a retrograde manner both along peripheral nerves, in the same way as ricin and abrin, and within the central nervous system [4,5]. This property could have different medical and biotechnological applications in the field of neuroscience to selectively lesion specific neurons, i.e., in behavior studies. The most toxic *Adenia* RIP is stenodactylin, which induces several molecular mechanisms triggering different cell death pathways [6,7]. Recently, kirkiin, a new type 2 RIP from the caudex of *Adenia kirkii*, was purified and characterized, showing a cytotoxicity comparable to that of stenodactylin. Kirkiin has N-glycosylase activity against mammalian and yeast ribosomes, and it is able to completely inhibit protein synthesis both in a cell-free system and in cells, and to induce cell death by apoptosis at very low doses in the human neuroblastoma cell line [8]. Due to its elevated cytotoxicity, it can be considered an attractive molecule for the production of immunotoxins for the treatment of cancers, and as a single agent for loco-regional treatments [7,9,10].

This study investigates the primary sequence of kirkiin, and a comparison with the sequences of other type 2 RIPs from *Adenia* species and ricin was performed, in order to provide useful information about the amino acids directly or indirectly involved in kirkiin toxicity. A three-dimensional structure was also predicted through homology modeling. Knowledge about the amino acid sequence associated with the structure analysis is essential to understand the protein function and to investigate structure–function relationships in the mechanism of action of kirkiin. Moreover, the carbohydrate-binding properties of kirkiin were here investigated in order to better understand the correlation between structure and function of the molecule. 

## 2. Results

### 2.1. Isolation and Cloning of Kirkiin Gene

**Figure 1:**
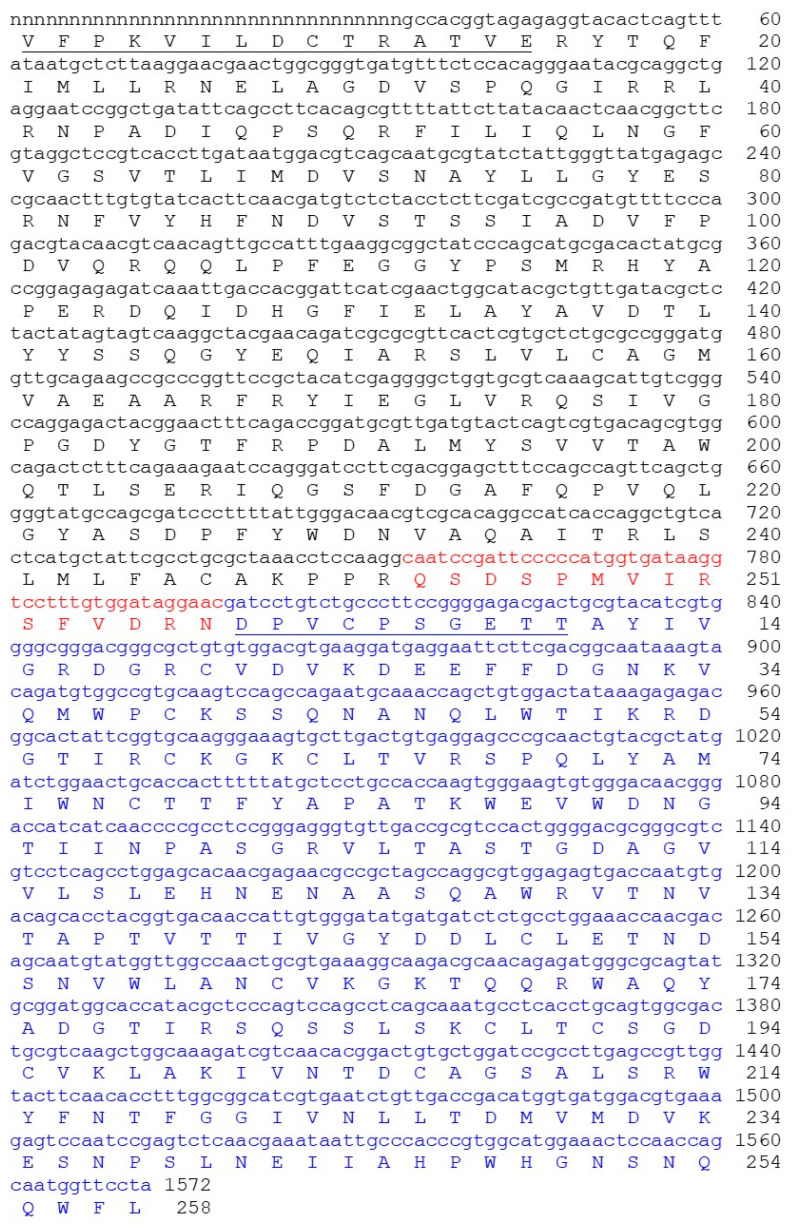
Full-length sequence and derived amino acid sequence of the kirkiin gene. The A chain is represented in black, the B chain is represented in blue, and the sequence of the linker peptide is represented in red. The N-terminal amino acid sequences of the A and B chains obtained by Edman degradation are underlined. Numbering refers to the position of the amino acids in the mature A and B chains. The DNA sequence for kirkiin was submitted to GenBank (accession number: OK283399). The letter “n” means “unknown nucleotide residue”, being the amino acid sequence obtained exclusively by Edman degradation.

The kirkiin gene sequence was determined by PCR amplification of *A. kirkii* genomic DNA. Based on the N- and C-terminal amino acid sequences of other RIPs derived from plants of *Adenia* genus (modeccin, lanceolin 1, lanceolin 2, stenodactylin, and volkensin) and on the information available in GenBank on volkensin amino acid sequence (CAD61022) and stenodactylin (MT580807), four specific primers were designed for PCR amplification of the kirkiin gene (see Section 4.2.2). Two primer pairs amplified two genomic DNA fragments corresponding to the A chain (A2-B1R) and the entire sequence of kirkiin (A2-B5R). The information obtained on the sequence was analyzed using the algorithms available on http://expasy.org (accessed on 15 October 2021) [11]. Excluding the nucleotide sequence coding for the signal peptide, the DNA sequence analysis revealed that kirkiin is encoded by a 1572 bp open reading frame (ORF) without introns, encoding a protein of 524 amino acids (Figure 1). The N-terminal sequence of kirkiin A chain was determined by direct Edman degradation and allowed us to obtain the sequence of the first 15 amino acid residues for the A chain (see below). As a control, also, the N-terminal sequence of the B chain was determined to confirm the method validity; the sequence deduced from the chemical sequencing perfectly matched the nucleotide sequence. The gene contains the 753 bp sequence encoding the A chain (251 amino acids with a theoretical molecular weight (Mw) of 28,324.21) and 774 bp sequence encoding the B chain (258 amino acids with a theoretical Mw of 28,506.13), which were separated by a sequence linker of 45 bp. The C-terminal end of the A chain and the linker sequence were estimated on the basis of the homology with other toxic type 2 RIPs from the same genus.

**Figure 2:**
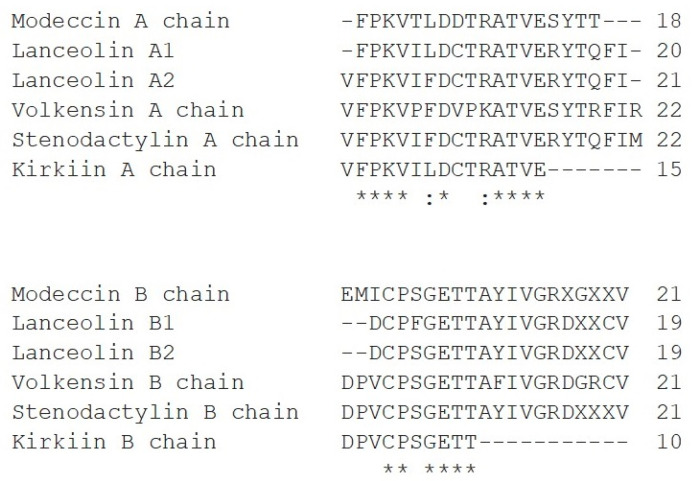
Amino acid alignment of the N-terminal sequences of modeccin, lanceolin, stenodactylin, volkensin, and kirkiin. Identical residues (*), conserved substitutions (:) are reported. X, unassigned amino acid positions [15].

The protein sequence alignment between the N-terminal sequences obtained for kirkiin and those already known for modeccin [12] (*A. digitata*), volkensin [13] (*A. volkensii*), lanceolin 1, lanceolin 2 (*A. lanceolata*), and stenodactylin (*A. stenodactyla*) [14] showed that kirkiin A chain shares 14/15 amino acids with stenodactylin and lanceolin A2. The different amino acid residue is Phe at position 7 in stenodactylin A chain, which is replaced by Leu in kirkiin A chain. This substitution is also present in the A chains of modeccin and in lanceolin 1. Moreover, all the N-terminal amino acid sequences of *Adenia* RIPs, except for volkensin, present a Cys residue at position 9 (position 8 for lanceolin A1). The identity among the B chains is very high, except for the first three N-terminal residues Asp-Pro-Val that are present in kirkiin as well as in volkensin and stenodactylin B chains but not in modeccin and lanceolin (Figure 2). 

**Figure 3:**
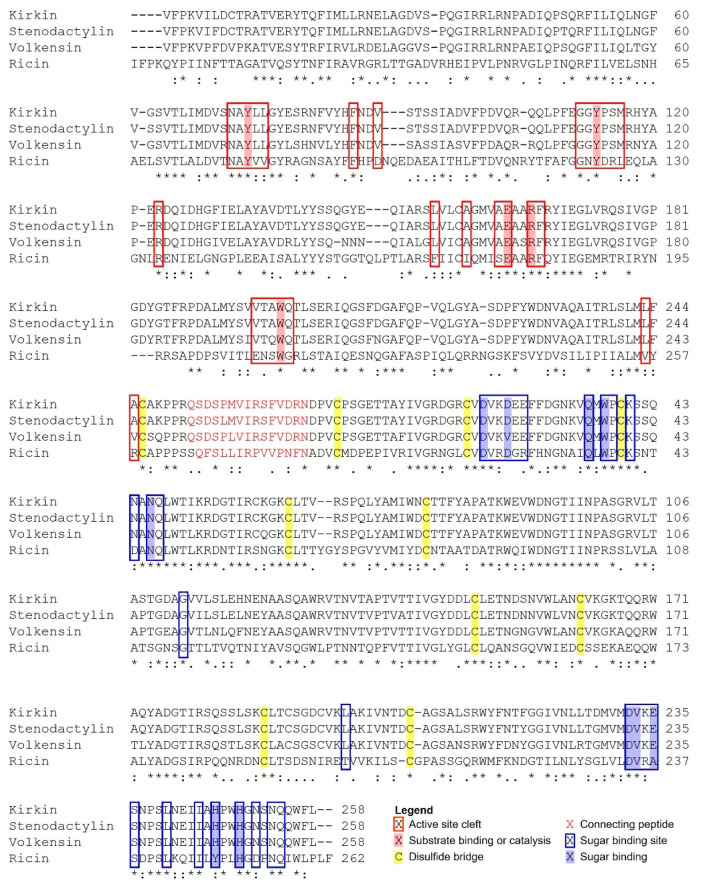
Alignment between kirkiin, stenodactylin (GenBank MT580807), volkensin (GenBank CAD61022), and ricin (GenBank P02879). Identical residues (*), conserved substitutions (:), and semiconserved substitutions (.) are reported. The A and B chains are written in black letters; the sequence of the linker peptide is in red. The putative amino acids that are present in the active site pocket (boxed in red) or in the galactoside-binding sites (boxed in blue), those involved in substrate binding or catalysis (highlighted in red), those involved in sugar binding (highlighted in blue), and those involved in disulfide bridges (highlighted in yellow) are represented, and they were assigned by comparison with the structure of ricin (PDB accession no. 2AAI, 3RTI, and 3RTJ). The dash indicates a gap introduced into the sequences to maximize alignments.

A multiple sequence alignment was performed between *Adenia* RIPs (kirkiin, stenodactylin, and volkensin) and ricin, which is the best-known RIP [16], using the program Clustal Omega [15] (Figure 3). The amino acid comparison showed a higher identity of kirkiin with RIPs purified from plants belonging to the same genus (96.3% with stenodactylin and 87% with volkensin) with respect to ricin (40.4%). The alignment between kirkiin and stenodactylin showed a higher identity of A chains (97.6%) in comparison to B chains (95.4%). On the contrary, the alignment between the kirkiin sequence and those of volkensin and ricin showed a higher identity of the B chains (89.2% with volkensin and 48.4% with ricin) with respect to the A chains (84.4% with volkensin and 35.1% with ricin). Similarly to stenodactylin, kirkiin contains a total of 15 cysteinyl residues, which is one more than that present in volkensin and four more than ricin. The kirkiin A chain includes three cysteinyl residues at the positions 9, 157, and 246. The B chain includes 12 cysteinyl residues (at positions 4, 20, 39, 59, 63, 78, 149, 162, 188, 191, 195, and 206). It is known for other type 2 RIPs, such as ricin [17], that the N-terminal cysteine of the B chain (Cys4) forms an interchain disulfide bridge with the cysteine at the C-terminal of the A chain (Cys246), and that eight cysteines in the B chain (Cys20-Cys39, Cys63-Cys78, Cys149-Cys162, and Cys188-Cys206) form conserved intramolecular disulfide bridges. Two possible glycosylation sites in the kirkiin B chain were identified by the program NetNGlyc1.0 [18] at position Asn93-Gly94-Thr95 and Asn133-Val134-Thr135. The amino acid residues reported to be involved in the active site of RIPs are conserved within the sequence of kirkiin and stenodactylin A chains, except for Ala199 and Ala245, which are replaced with Gln198 and Val244 in volkensin. All the amino acids present in sugar binding sites are conserved in the kirkiin B chain, similarly to stenodactylin and volkensin (Figure 3).

### 2.2. Structural Analysis of Kirkiin

**Figure 4:**
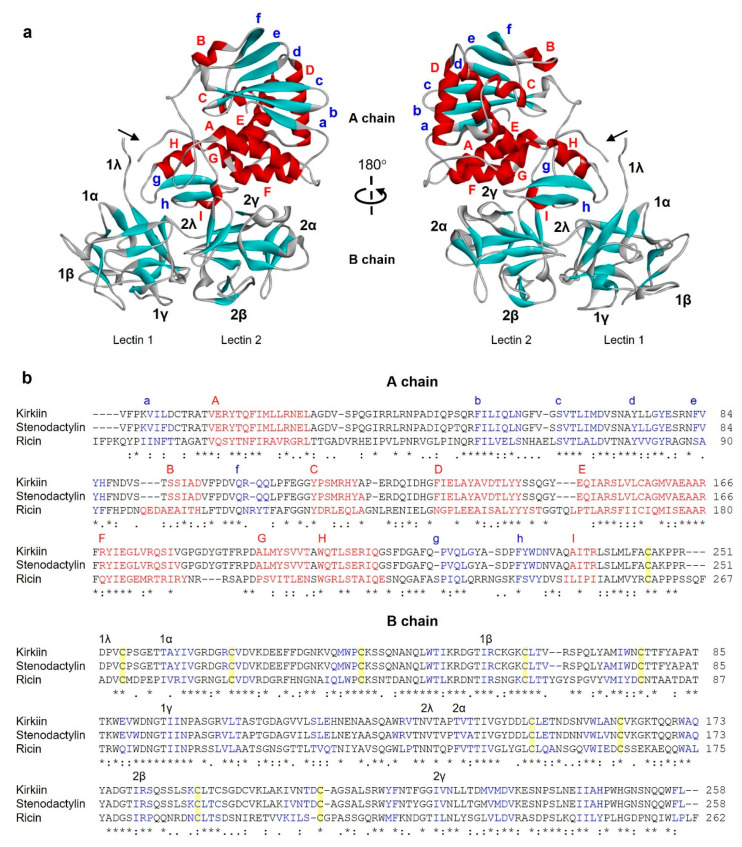
Structure of kirkiin. (**a**) Three-dimensional structure of kirkiin. The three-dimensional structural modeling was carried out on the I-TASSER server, and the figure was generated using Discovery Studio 2016. The α helices (red), the β chains (cyan), and the coils (gray) are represented. Arrows indicate the position of the disulfide bond linking A and B chains. (**b**) Amino acid sequence alignment of the A and B chains of kirkiin, stenodactylin, and ricin [22]. The strands (blue), the helices (red), and the cysteines involved in the disulfide bonds (highlighted in yellow) are indicated. The helices are labeled from A to I, and the strands of the β sheets are labeled from a to h in the A chain. The structural subdomains in the B chain are also indicated. Identical residues (*), conserved substitutions (:), and semiconserved substitutions (.) are reported.

Given the availability of the complete amino acid sequence, it was possible to predict the three-dimensional structure of kirkiin with a computational model using several type 2 RIP crystal structures as templates. The best three-dimensional model obtained for kirkiin is shown in Figure 4a, and it was found to have a confidence score (C-score) of 0.61, template modelling (Tm) score of 0.80 ± 0.09, and root-mean-square deviation (RMSD) of 6.1 ± 3.8 Å, which satisfied the range of parameters for molecular modeling. The overall folding of type 2 RIPs is conserved in kirkiin, apart from a few discrepancies due to some deletions and insertions in loop regions. The kirkiin A chain structure consists of three folding domains. The first domain includes the N-terminus until the residue Phe109, with the first four residues not structured. It is composed of six antiparallel β-sheets (strands from a to f) and two α-helices (helices A and B) alternating in the order aAbcdeBf (Figure 4b). Tyr74, one of the amino acids directly involved in the binding of adenine, is located in the first domain. The second domain extends from Glu110 to Ala199 and consists of five α-helices (helices from C to G), with a classical helix–loop–helix motif. The second amino acid involved in adenine binding (Tyr113) and the catalytic amino acids (Glu163 and Arg166) are located here. The last domain consists of two α-helices and two antiparallel β-sheets in a α-helix–β-fork–α-helix motif (HghI), ending with an unstructured coil region in the C-terminus. This structural motif seems to be important for the interaction of RIPs with cell membranes [19] and for the explanation of their biological and toxic activities [20], and it contains the residue Trp200 of the active site. The three folding domains of the A chain form a deep pocket which accommodates the conserved active site. Similar to other type 2 RIPs, the kirkiin B chain is made of two homologous globular lectin domains arisen by gene duplication [21], which is exclusively formed by β-sheets. Each domain consists of four homologous subdomains (1λ, 1α, 1β, and 1γ for lectin 1; 2λ, 2α, 2β, and 2γ for lectin 2). The subdomains 1λ (from the B chain N-terminus to residue Thr9) and 2λ (residues Val131 to Pro137) are responsible for the binding to the A chain and for the interconnection between the two B chain domains, respectively. The subdomains 1α (residues Thr10 to Thr56), 1β (residues Ile57 to Gly94), and 1γ (residues Thr95 to Asn130) are arranged in a trefoil structure. This arrangement is also present in lectin 2 with subdomains 2α (residues Thr138 to Thr178), 2β (residues Ile179 to Gly221), and 2γ (Ile222 to Leu258) (Figure 4b). The subdomains 1α and 2γ contain the two galactose binding sites.

### 2.3. Carbohydrate Binding Properties of Kirkiin

Kirkiin showed hemagglutination activity (HA) on human A, B, and 0 erythrocytes being the minimum concentration required for activity of 0.175 mg/mL for both the A and 0 blood groups, and of 0.35 mg/mL for the B blood group (data not shown). To understand the sugar binding specificity of kirkiin, the inhibition of hemagglutination was carried out with several monosaccharides and disaccharides (Table 1). The results showed that the HA of kirkiin was inhibited by D-galactose and its derivative lactose at very similar concentrations (0.012 and 0.011 M, respectively). No affinity for D-glucose, D-fructose, D-mannose, D-sorbitol, D-mannitol, L-fucose, N-Acetyl-D-mannosamine, and sucrose was observed by kirkiin at the maximum sugar concentration tested. D-galactose was able to inhibit kirkiin HA at concentration one titer lower than that of stenodactylin (0.023 M), while lactose has the same inhibiting power for both *Adenia* RIPs. Lactose was a better inhibitor than D-galactose of stenodactylin HA, which is in agreement with previous results [14]. Lactose and D-galactose were also the best inhibitors of ricin HA; in this case, the HA inhibition was observable at very low concentrations, 2–3 titers (D-galactose) and 5 titers (lactose) lower than those of kirkiin and stenodactylin, respectively. Very low affinity was observed with D-glucose, D-fructose, D-mannose, D-sorbitol, and L-fucose in agreement with previous results [23].

### 2.4. Molecular Docking

As shown in Figure 3, the sequences of the sugar binding sites of kirkiin are similar to those of stenodactylin and volkensin. The amino acids in the pockets of 1α and 2γ sites are identical in all three proteins with the exception of Asp25 of kirkiin and stenodactylin, which is Val25 in volkensin. This agrees with the finding that kirkiin and stenodactylin have similar affinities for D-galactose and lactose (Table 1). However, there are differences with ricin sugar-binding sites. So, at the 1α site, there are 10 identical amino acids out of the 14 that compose the binding pocket [22], that is 71.4%, while in the 2γ site, there are eight identical amino acids out of the 13 that make up the binding pocket, that is 61.5%. It should be noted that all the amino acids involved in binding to sugars at the 1α site are conserved in kirkiin and ricin, while at the 2γ site, Glu235 and His246 in kirkiin change to Ala237 and Tyr248 in ricin, respectively (Figure 3). These changes could explain the large differences in lactose and D-galactose affinities between kirkiin and stenodactylin and ricin (Table 1). In fact, the affinity of ricin for D-galactose is four and eight times higher than those of kirkiin and stenodactylin, respectively, and the affinity of ricin for lactose is 16 times greater than that of kirkiin or stenodactylin.

This prompted us to study how D-galactose binds to kirkiin 1α and 2γ sites in comparison to ricin, since glucose does not bind to kirkiin, and therefore, lactose has to bind to this protein via galactose as with ricin. For this purpose, we perform a comparative molecular docking study using Autodock 4.2. Using the sequence of the kirkiin B chain where the first nine amino acids, corresponding to subdomain 1λ were excluded, a structure was obtained by comparative modeling in the I-Tasser server that presented better values of C-score (1.32), Tm score (0.90 ± 0.06), and RMSD (3.2 ± 2.2 Å) than the model obtained with the whole protein. Docking was performed with D-galactose and lactose, and solutions matching the two structures were chosen. As shown in Figure S2, with the 1α site of ricin, there are no differences between the results obtained by molecular docking with D-galactose or lactose and those obtained by crystallography and X-ray diffraction, while small differences are observed at the 2γ site, although the orientation of the pyranosic ring is identical.

**Figure 5:**
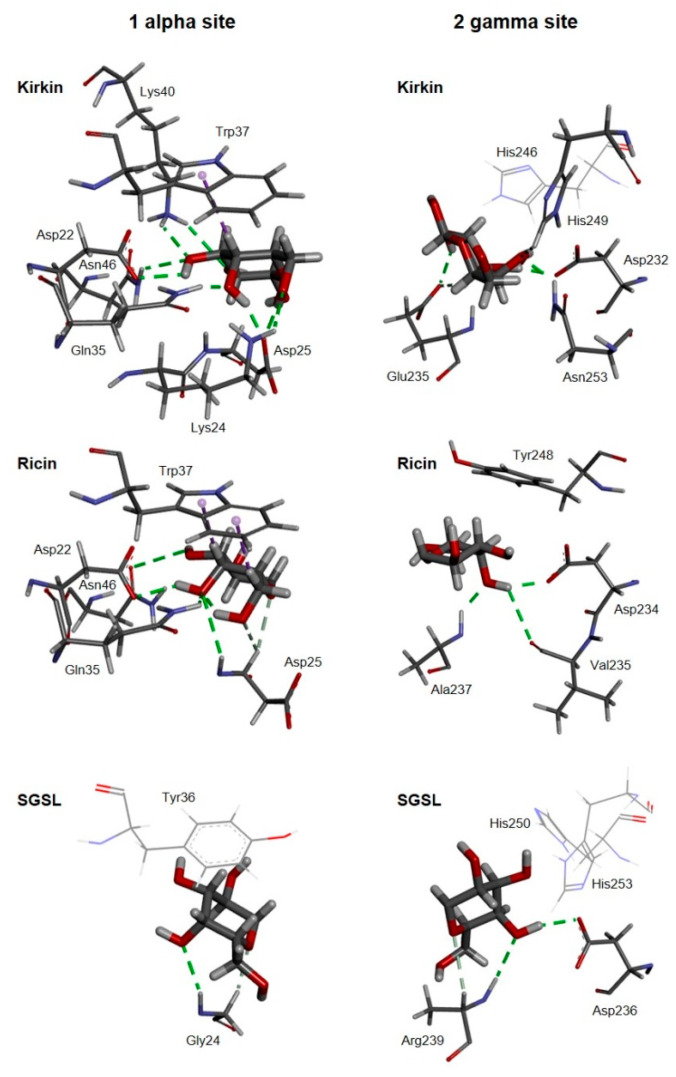
Three-dimensional models of the galactose-binding sites from kirkiin, ricin, and SGSL. The galactose-binding sites from either kirkiin, ricin (PDB 2AAI), and SGSL (PDB 5Y97) complexed with β-d-galactopyranose (thick sticks) are represented. The amino acids that bind the galactose molecule by either C–H–π interactions (dashed purple lines) or both conventional (dashed green lines) and non-conventional (dashed light green lines) hydrogen bonds are represented by thin sticks. His246 from kirkiin, and Tyr36, His250, and His253 from SGSL are also represented (lines).

As shown in Figure 5, the amino acids involved in D-galactose binding at the 1α site of ricin are conserved in kirkiin, and the way D-galactose binds is very similar in both proteins. In both cases, the binding of β-D-galactopyranose is the result of the C–H–π interaction between the aromatic ring of Trp37 and the apolar face of the pyranosic ring of galactose. The polar groups of the polar face of galactose form hydrogen bonds with the amino acids Asp22, Asp25, Gln35, and Asn46, and, in the case of kirkiin, the hydrogen bonds can also be formed with Lys24 and Lys40. In snake gourd seed lectin (SGLS), a non-toxic type 2 RIP obtained from seeds of *Trichosanthes cucumerina* L. (= *Trichosanthes anguina* L.), the apolar face of the pyranosic ring of galactose binds to the aromatic ring of Tyr36, while the polar face forms hydrogen bonds with the amino acids on the other side of the pocket of the 1α site, mainly with Gly24 [24] (Figure 5).

Unlike the 1α site, the 2γ site of kirkiin is very different from that of ricin. In ricin, the aromatic ring of Tyr248 is oriented toward the apolar face of the pyranosic ring, being able to establish a C–H–π interaction with the apolar face of it. The polar face of D-galactopyranose establishes hydrogen bonds, on the other side of the pocket of the 2γ site, with the amino acids Asp234, Val235, and Ala237 (Figure 5). In kirkiin (as in stenodactylin), Tyr248 is replaced by His246. Unlike ricin, the apolar face of galactose is not oriented toward the aromatic ring and there can be no C–H–π interaction between galactose and histidine. The binding is reached by hydrogen bonds with Asp232, Glu235, His249, and Asn253. This arrangement is similar to that presented at the 2γ site of the SGSL, where the apolar face is not oriented toward His250, and the polar face forms hydrogen bonds with Asp236 and Arg239 (Figure 5).

## 3. Discussion

Kirkiin, a type 2 RIP isolated from *A. kirkii* caudex, is one of the most potent plant toxins known, with a cytotoxicity comparable to that of stenodactylin and ricin [8]. The aim of the present work was to determine the complete amino acid sequence of kirkiin as well as to predict the protein structure by using computational homology modeling. 

Sequence analysis showed that kirkiin is encoded by a 1572 bp gene without introns, as previously reported for other RIPs, such as volkensin [13], ricin [25], abrin [26], and viscumin [27]. Kirkiin shares a high degree of identity with the type 2 RIPs stenodactylin and volkensin, since all toxins are phylogenetically related. This expected high identity also explains why kirkiin highly cross-reacted with serum against stenodactylin and volkensin [8]. The N-terminal sequences of kirkiin A and B chains were found to be identical to those of stenodactylin, with the exception of the residue Phe in position 7 in the stenodactylin A chain, which is replaced by Leu in kirkiin A chain. This substitution is also present in the A chains of modeccin and the isoform 1 of lanceolin. Interestingly, the N-terminal sequence of kirkiin A chain shares the presence of an additional cysteine residue with the A chains of other *Adenia* RIPs, except for volkensin. It could be of interest to better investigate in the future the role of this cysteine residue, since the reduction of the disulfide bridge inside the cell is important for type 2 RIP toxicity.

The complete amino acid sequence showed that kirkiin, similar to stenodactylin, contains the highest number of cysteine residues among type 2 RIPs. It is known for the type 2 RIPs that the C-terminal cysteine of the A chain forms an interchain disulfide bridge with the cysteine at the B chain N-terminus [28]. Similarly, Cys246 of the kirkiin A chain is involved in a disulfide bridge with Cys4 of the B chain. The type 2 RIP B chain consists of two lectin domains, each organized around two disulfide bridges [29]. This scheme is also present in kirkiin (Cys20 to Cys39 and Cys63 to Cys78 for lectin 1 of B chain; Cys149 to Cys162 and Cys188 to Cys206 for lectin 2 of B chain). Three other cysteine residues are included in the kirkiin B chain (Cys59, Cys191, and Cys195). In the 3D model, Cys59 seems to be isolated in lectin 1, while Cys191 and Cys195 are located in a loop in lectin 2, which is close enough to form a disulfide bridge. This pattern was already observed in the volkensin 3D model [13]; however, the role of these cysteine residues is still unknown. 

Two glycosylation sites are located in the kirkiin B chain. The presence of carbohydrates could explain the difference in the molecular weight of the B chain determined on the basis of the amino acid sequence (28.5 kDa) and on that observed by electrophoretic mobility (35 kDa) [8]. The glycosylation level of RIPs has proven to be important in explaining their toxic activity. It can influence the protein structure, impacting either the overall structure or the local conformation, and consequently, it can affect RIP intracellular transport to the endoplasmic reticulum and to other compartments, thus influencing its cytotoxicity [30,31]. 

A molecular model of kirkiin has been elaborated on the basis of the crystallographic coordinates of ricin, which shares a high sequence homology with kirkiin. Knowledge about the amino acid sequence associated with the structure analysis of the RIP is essential to understand the protein function and to correlate structural differences to the cytotoxic mechanisms of RIPs. The three-dimensional model obtained for kirkiin revealed that it shares the general structure of type 2 RIPs. The amino acids of the active site, responsible for the enzymatic mechanism of RIPs [32], are also conserved in kirkiin: Tyr74, Tyr113, and Trp200 are the amino acids directly involved in substrate binding, whereas Glu163 and Arg166 are the amino acids responsible for the catalysis. The highly conserved Phe167 located in the active site is also present in kirkiin; its function is still unclear but seems to be involved in stabilizing the conformation of the side chain of Arg166 [33]. Most of the additional residues participating in the active site of ricin (Asn78, Arg134, Gln173, Ala178, Glu208, and Asn209) are also conserved in kirkiin. They are involved in the stabilization of the active site, and almost all are conserved among the A chains of type 2 RIPs [34,35]. Most of these amino acids are conserved in the kirkiin A chain (Asn72, Arg123, and Ala164), except for Gln173, Glu208, and Asn209 in ricin that are replaced in kirkiin by Gly159, Val197, and Thr198, respectively. These substitutions are also present in the stenodactylin [22] and volkensin A chains [13]. In addition, Ser204, located close to the active site and evolutionarily conserved among RIPs with the function of stabilizing the conformation of the side chain of Trp200 [36], is also conserved in kirkiin. Almost all the amino acids involved in sugar binding in the 1α and 2γ subdomains of the ricin B chain [37] are conserved in kirkiin (Asp22, Asp25, Gln35, and Trp37 for the 1α subdomain and Asp232, Ile244, Asn253, and Gln254 for the 2γ subdomain). Two exceptions in the 2γ subdomain were identified for Ala237 and Tyr248 in ricin, which are replaced with Glu235 and His246 in kirkiin, respectively. The same substitutions were observed in stenodactylin [22] and volkensin [13]. In particular, the presence of His instead of Tyr was also identified in *R. communis* agglutinin [38] and in PMRIPm of *Polygonatum multiflorum* [35]. A previous study demonstrated that the substitution of Tyr248 with His in the ricin B chain introduced a positive charge in the 2γ subdomain, preventing the interaction between the pyranose ring of galactose and the aromatic ring of Tyr. This change caused a reduction in the binding activity of ricin [39]. Moreover, the presence of the aromatic residue Phe249 in ebulin l from *Sambucus ebulus,* instead of Tyr248 in ricin, results in a deficient sugar binding and a consequently lower RIP cytotoxicity [40]. These data suggest that the sugar affinity is essential to explain the biological activity of RIPs. The recognition and binding to exposed galactose residues on cell membrane is the first step in RIP–cell interaction. Thus, small differences in sugar binding might affect RIP cytotoxic activity. Most of the RIPs have galactose/N-acetylgalactosamine (gal/galNAc) affinity, but some of them can also show different sugar specificity. For example, *Sambucus nigra* agglutinin I has affinity for both gal/galNAc and sialic acid [41]. Mistletoe lectin I has specificity for galactose, L-arabinose, and poor affinity for sialic acid [42]. Changes in the 2γ subdomain of the *Sambucus* tetrameric RIPs (Glu235 by Gln, His246 by Tyr, and His249 by Thr or Asn) cause specificity toward galactose and N-acetyl neuraminic acid [43]. Moreover, changes in both 1α and 2γ binding sites of *Iris hollandica* RIPs (Trp37 by Ser and His246 by Trp) are responsible for specificity toward mannose [44]. In the case of kirkiin, the hypothesis that changes in the 2γ site could affect cell binding does not correlate with the high cytotoxicity that kirkiin has shown in previous studies [8]. For this reason, we considered it interesting to study the sugar affinity properties of kirkiin in order to have more detailed information that would help us understand its unique properties. Hemagglutination inhibition assay showed that kirkiin and stenodactylin have similar affinities for D-galactose and lactose (Table 1), which is probably due to the high sequence identity of the sugar binding sites. Nevertheless, the affinity of kirkiin for these sugars was lower with respect to ricin. According to docking experiments, both the 1α and 2γ sites of kirkiin can bind lactose and D-galactose. The 1α sites of both ricin and kirkiin are identical. The interaction of tryptophan with the apolar face of D-galactopyranose allows the formation of numerous hydrogen bonds between the polar face and the amino acids on the other side of the 1α site pocket. It is worth noting that the orientation of the C-4 hydroxyl group of D-glucopyranose toward the aromatic rings would prevent this type of binding. In SGSL, the binding to the 1α site is very different [24]. The lack of toxicity of SGLS has been attributed to the result of a combination of changes in the active site of the A chain (it does not bind adenine) and the sugar binding sites of the B chain. In SGLS, the aromatic ring of Tyr36 could play the same role as tryptophan in kirkiin and ricin, and the polar face of the pyranosic ring could form hydrogen bonds with the amino acids on the other side of the pocket of the 1α site, mainly with Gly24 (Figure 5), but also with other amino acids. However, this binding would be weak, and for this reason, the 1α site can bind D-galactose but cannot retain it [24]. 

Since the kirkiin 1α site is identical to that of ricin, the two amino acid substitutions in the 2γ site, in particular the replacement of Tyr248 of ricin with His246, are evidently sufficient to lower the affinity of kirkiin for these sugars. These changes cause a different arrangement within the pocket of the 2γ site with respect to ricin, which could justify the low affinity for lactose and D-galactose. This arrangement is similar to that of the 2γ site of SGSL, which is able to bind and retain galactose [24].

All these data show that kirkiin has a high degree of identity with RIPs from *Adenia* genus plants as well as a structure that preserves the overall folding of type 2 RIPs. 

Despite the substantial difference in the structure of the 2γ site with respect to ricin that may explain the lower affinity for sugars, kirkiin and *Adenia* RIPs are the most toxic plant proteins [8]. Therefore, their biological properties, especially cytotoxicity, could be correlated to other mechanisms that overcome the differences in cell binding. The high cytotoxic potential of kirkiin and its ability to elicit different cell death pathways makes kirkiin a suitable candidate as a pharmacological tool for drug targeting. The high toxicity of native kirkiin would allow its use only for loco-regional treatments. However, the kirkiin A chain could be linked to carriers targeting cancer cells in systemic therapy. Further studies will be useful to better clarify the modalities and the types of triggered cell death and the consequent biological behavior of kirkiin in vivo.

## 4. Materials and Methods

### 4.1. Materials

Kirkiin was purified from the caudex of *Adenia kirkii* as described by Bortolotti et al. [8]. *Adenia* plants volkensin and stenodactylin were purchased from Exotica Botanical Rarities, Erkelenz-Golkrath, Germany, while *A. kirkii* was purchased from Mbuyu–Sukkulenten, Bielefeld, Germany. If not used immediately on arrival, the plants were kept in the greenhouse of the Botanical Garden of the University of Bologna. 

Genomic DNA from *A. kirkii* was extracted through DNeasy Minikit (Qiagen Iberia SL, Barcelona, Spain). Primers were synthesized by Integrated DNA Technologies (Leuven, Belgium). Taq Polymerase was obtained from Biotools B&M Labs S.A. (Madrid, Spain). PCR products were purified using the NucleoSpin^®^ Gel and PCR Clean-up kit (Macherey-Nagel GmbH & Co KG, Düren, Germany). Molecular cloning of PCR products was carried out using TA Cloning^®^ Kit Dual Promoter (Invitrogen-Thermo Fisher Scientific, Alcobendas, Spain). Plasmids were sequenced by CENIT Support system (Villamayor, Salamanca, Spain).

### 4.2. Methods

#### 4.2.1. Isolation of DNA

The caudex of *A. kirkii* was disrupted using a mortar and pestle and grinded to a fine powder under liquid nitrogen and total DNA was extracted through DNeasy Minikit (Qiagen), according to the manufacturer’s instruction. Then, 1.2 μg of total DNA was obtained from 100 mg of frozen tissue. The DNA content was determined by a Beckman DU 640 Spectrophotometer (Beckman, Brea, CA, USA).

#### 4.2.2. Primer Design for PCR Amplification

Gene-specific primers for the full-length kirkiin sequence were designed and synthesized based on volkensin and stenodactylin amino acid sequences (CAD61022 and MT580807) and N-terminal sequences available for *Adenia* RIPs. Four oligonucleotides were designed for PCR amplification of the kirkiin gene: A2 for N-terminal sequence of the A chain; B1 and B1 reverse (B1R) for N-terminal sequence of the B chain, and B5 reverse (B5R) for the C-terminal end of the B chain. The sequences of the primers are reported in Table 2.

#### 4.2.3. Amino Acid Sequencing by Edman Degradation

Kirkiin was blotted both in reduced and non-reduced form onto PVDF membrane (Immobilon P membrane) in 50 mM sodium borate, pH 9.0/20% methanol/0.1% SDS at 1 mA/cm^2^ PVDF membrane for 2–3 h at 4 °C. The protein band was stained by Ponceau Red (0.5% Ponceau S, 1% acetic acid in Milli-Q water). The blotted bands that corresponded to kirkiin A and B chains (50 µg each) were cut and directly subjected to N-terminal automated protein sequencing using the PPSQ–33B sequencer (Shimadzu Corporation, Tokyo, Japan). Edman degradation was performed by Protein and Peptide Sequencing Service—Institute of Biosciences and Bioresources (National Research Council, Naples).

#### 4.2.4. Gene Amplification and Cloning

Genomic DNA was used as a template for PCR amplification in order to determine the amino acid sequence of kirkiin. PCR was conducted using the thermal cycler Gene Amp PCR system 2400 (Perkin Elmer, Waltham, MA, USA). The PCR reaction for gene amplification included 40 ng of total DNA, 0.5 μM of each primer, PCR buffer/Mg^2+^ (Tris HCl 75 mM pH 9.0, KCl 50 mM, (NH_4_)_2_SO_4_ 20 mM, MgCl_2_ 2 mM), 0.25 mM dNTPs Mix, and 0.5 U/μL Taq Polymerase (Biotools). PCR amplification was carried out with the following conditions: an initial denaturation at 94 °C for 3 min, followed by 40 cycles of 94 °C for 30 s, 55 °C for 45 s, and 72 °C for 2 min. Three couples of primers were used to detect the full-length amino acid sequence of kirkiin (A2–B1R for the A chain and part of the B chain, B1–B5R for the B chain; A2–B5R for the complete sequence). The amplified fragments were analyzed by agarose gel electrophoresis, showing the expected size of about 0.8 kb for the A chain with part of the B chain (Figure S1A) and about 1.6 kb for the entire sequence (Figure S1B). B1–B5R failed to amplify the target region. PCR products were purified using the NucleoSpin^®^ Gel and PCR Clean-up kit (Macherey-Nagel), according to the manufacturer’s instruction. The two purified amplicons were ligated into the pCR^®^II vector (TA Cloning^®^ Kit Dual Promoter, Invitrogen) and then were used to transform the highly competent *E. coli* InVαF’ cells. The purified plasmids were sequenced by CENIT Support system. The information obtained on the sequence was analyzed using the algorithms available on http://expasy.org (accessed on 15 October 2021) [11].

#### 4.2.5. Sequence Retrieval and Alignment

The sequences of stenodactylin (Accession number MT580807) and volkensin (Accession number Q70US9) are available in the National Center for Biotechnology Information (NCBI) sequence database (https://www.ncbi.nlm.nih.gov/protein/ (accessed on 15 October 2021)). Sequence alignment was performed using the Clustal Omega server (https://www.ebi.ac.uk/Tools/msa/clustalo/ (accessed on 15 October 2021)) [16]. Glycosylation sites were predicted using the NetNGlyc1.0 server [18].

#### 4.2.6. Protein Structure Studies and Graphical Representation

The structures of ricin (accession numbers 2AAI, 3RTI, and 3RTJ) and SGSL (accession number 5Y97) are available in the Protein Data Bank (https://www.rcsb.org/ (accessed on 15 October 2021)). Three-dimensional structural modeling of kirkiin was carried out on the I-TASSER server (https://zhanglab.ccmb.med.umich.edu/I-TASSER/ (accessed on 15 October 2021)) [45]. Study and graph representations of protein structures were performed with the aid of the Discovery Studio Visualizer suite (v16.1.0) (https://www.3dsbiovia.com/ (accessed on 15 October 2021)).

#### 4.2.7. Hemagglutination Activity and Carbohydrate-Binding Properties 

Hemagglutination activity (HA) was assayed using 2% human erythrocyte suspension collected from voluntary donors (0+, A+, and B+). Blood samples were collected in phosphate-buffered saline (PBS) and centrifuged at 500× *g*. The erythrocyte pellet was washed and resuspended in the same buffer to make 2% red blood cell suspension. The HA was determined in microtiter plates. Each well contained 50 µL of serial dilutions of the proteins and 50 µL of erythrocyte suspension and the plates were incubated for 1 h at room temperature. The minimum concentration of protein causing complete agglutination was visually evaluated. For hemagglutination inhibition assay, ten sugars (D-glucose 3.2 M, D-galactose 1.5 M, D-Fructose 3.2 M, D-Mannose 3.0 M, D-Sorbitol 2.9 M, D-Mannitol 0.9 M, L-Fucose 1.1 M, N-Acetyl-D-mannosamine 6.8 M, Lactose 0.7 M, Sucrose 1.2 M) were tested for their ability to inhibit the HA of the RIPs with 0+ blood group. Each well contained 25 µL of carbohydrates serially diluted and an equal volume of the RIP at a concentration one titer higher than the HA dose. An equal volume of erythrocyte suspension (50 µL) was added to each well and incubated for 1 h at room temperature. The maximum concentration of the tested sugars that completely inhibited HA activity was determined.

#### 4.2.8. Molecular Docking

The structures of beta-D-galactose (PubChem CID 439353) and beta-lactose (PubChem CID 6134) are available in the PubChem database (https://pubchem.ncbi.nlm.nih.gov/ (accessed on 15 October 2021)) [46]. Docking was carried out using Autodock 4.2 (http://autodock.scripps.edu/ (accessed on 15 October 2021)), as previously described [47]. Docking of d-galactose was performed on a grid of 120 × 120 × 120 points, with the addition of a central grid point. The grid was centered on the C4 of the galactose of either the 1α site or the 2γ site of the 2AAI ricin structure. Grid spacing was 0.125 Angstroms, leading to a grid of 15 × 15 × 15 Angstroms. For each molecule, 100 docking runs were performed. The generated 100 docking poses were clustered by root mean square (RMS) difference with a cutoff value of 0.5 Angstroms for each case. The top-ranked pose of the most populated clusters was retained and further analyzed with the Discovery Studio Visualizer suite (v16.1.0). The docking of beta-lactose was performed as indicated for d-galactose but using a grid of 124 × 124 × 124 points and a grid spacing of 0.180 Angstroms, leading to a grid of 22.32 × 22.32 × 22.32 Angstroms. The generated 100 docking poses were clustered by RMS difference with a cutoff value 2.0 Angstroms for each case. The top-ranked pose of the most populated clusters was retained and further analyzed with the Discovery Studio Visualizer suite (v16.1.0). Finally, the results obtained with d-galactose and beta-lactose were matched, and the coinciding solutions were selected.

## 5. Conclusions

The knowledge of amino acid sequence and the 3D structure prediction of kirkiin represent essential tools because of the potential use of kirkiin in medicine, for cancer treatment, and of its biotechnological applications in neuroscience. Moreover, the comparison between the structural properties of kirkiin and those of other type 2 RIPs is useful for explaining the differences in enzymatic activity and toxicity.

## Figures and Tables

**Table 1:** Inhibition of the hemagglutination activity of kirkiin, stenodactylin, and ricin by sugars.

Carbohydrates	Minimum Concentration Inhibiting Hemagglutination (M)		
	Kirkiin	Stenodactylin	Ricin
D-glucose	NI	1.6	1.6
D-galactose	0.012	0.023	0.0029
D-fructose	NI	NI	1.6
D-mannose	NI	NI	1.5
D-sorbitol	NI	NI	1.45
D-mannitol	NI	NI	NI
L-fucose	NI	NI	0.28
N-Acetyl-D-mannosamine	NI	NI	NI
Lactose	0.011	0.011	0.00068
Sucrose	NI	NI	NI

NI = no inhibition of hemagglutination at the maximum sugar concentration tested.

**Table 2:** Primer sequences.

Primer	Sequence
A2	5′ GCCACGGTAGAGAGRTACACT 3′
B1R	5′ AAGTCGTCTCCCCGGAAGGGC 3′
B1	5′ TGCCCTTCCGGGGAGACGACT 3′
B5R	5′ TAGGAACCATTGCTGGTTGGA 3′

## Data Availability

The DNA sequence for kirkiin was submitted to GenBank (accession number: OK283399).

## Supplementary Materials

The following are available online at https://www.mdpi.com/article/10.3390/toxins13120862/s1: Figure S1: Amplification of kirkiin A chain with part of the B chain (A, lane 1), and the entire sequence (B, lane 1). Mw: λ Hind III/EcoRI double digest DNA marker. The red squares indicate the amplification products. Figure S2: Comparison of the three-dimensional models of ricin (PDB 2AAI) sugar-binding sites 1 alpha (left) and 2 gamma (right) bound to D-galactose. The results obtained using AutoDock 4.2 with either β-D-galactopyranose (green lines, PubChem CID 439353) and β-lactose (blue lines, PubChem CID 6134) are compared with those obtained by X-ray diffraction (gray lines) as reported previously [48]. The amino acids of the sugar-binding sites are represented by sticks. In the case of β-lactose, only the galactose part is represented.
