# Supplementary material for: Sequence, Structure, and Binding Site Analysis of Kirkiin in Comparison with Ricin and Other Type 2 RIPs
Source: Toxins (Basel). 2021 Dec 3;13(12):862. doi: 10.3390/toxins13120862 (PMC8705660; doi:10.3390/toxins13120862)
Supplement: Supplementary file 1 [file toxins-13-00862-s001.zip › toxins-1453128-supplementary.pdf]

Supplementary Materials: Sequence, Structure and Binding Site Analysis of Kirkiin in Comparison with Ricin and Other type 2 RIPs

Stefania Maiello, Rosario Iglesias, Letizia Polito, Lucía Citores, Massimo Bortolotti, José M. Ferreras and Andrea Bolognesi

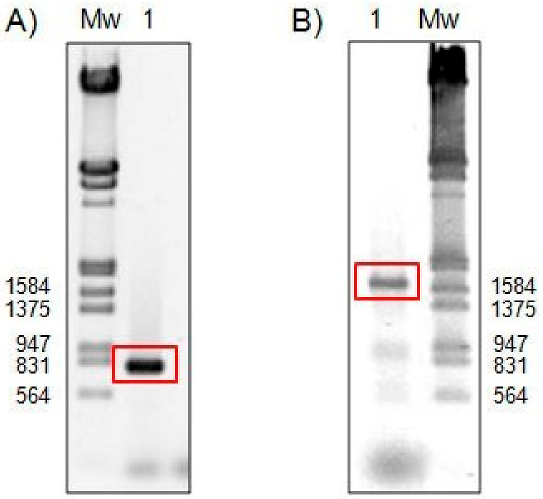

Figure S1. Amplification of kirkiin A-chain with part of the B-chain (A, lane 1), and the entire sequence (B, lane 1). Mw: λ Hind III/EcoRI double digest DNA marker. The red squares indicate the amplification products.

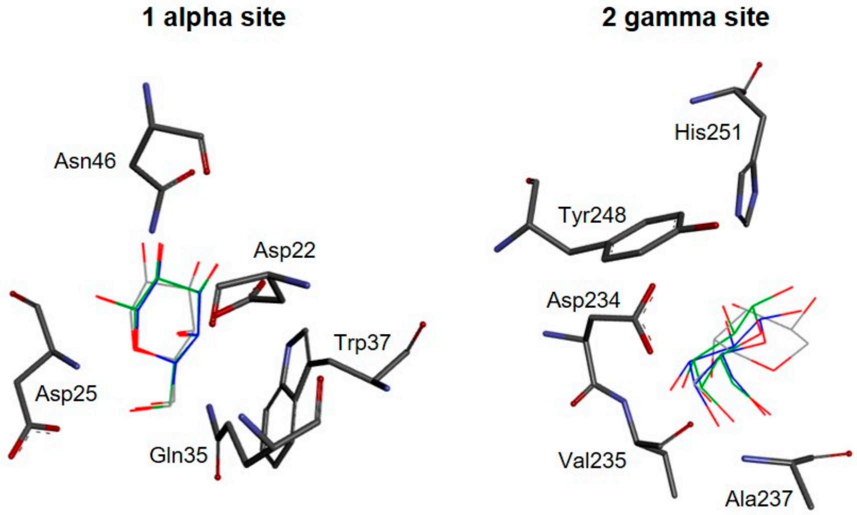

Figure S2. Comparison of the three-dimensional models of ricin (PDB 2AAI) sugar-binding sites 1 alpha (left) and 2 gamma (right) bound to D-galactose. The results obtained using AutoDock 4.2 with either β-D-galactopyranose (green lines, PubChem CID 439353) and β-lactose (blue lines, PubChem CID 6134) are compared with those obtained by X-ray diffraction (grey lines) as reported previously [48]. The amino acids of the sugar-binding sites are represented by sticks. In the case of β-lactose, only the galactose part is represented.
